# Analysis of Glass-Reinforced Epoxy Material for Radio Frequency Resonator

**DOI:** 10.1155/2014/831435

**Published:** 2014-04-06

**Authors:** M. R. Zaman, M. T. Islam, N. Misran, Baharudin Yatim

**Affiliations:** ^1^Institute of Space Science (ANGKASA), 43600 UKM Bangi, Malaysia; ^2^Department of Electrical, Electronic and Systems Engineering, Universiti Kebangsaan Malaysia, 43600 UKM Bangi, Malaysia

## Abstract

A radio frequency (RF) resonator using glass-reinforced epoxy material for C and X band is proposed in this paper. Microstrip line technology for RF over glass-reinforced epoxy material is analyzed. Coupling mechanism over RF material and parasitic coupling performance is explained utilizing even and odd mode impedance with relevant equivalent circuit. Babinet's principle is deployed to explicate the circular slot ground plane of the proposed resonator. The resonator is designed over four materials from different backgrounds which are glass-reinforced epoxy, polyester, gallium arsenide (GaAs), and rogers RO 4350B. Parametric studies and optimization algorithm are applied over the geometry of the microstrip resonator to achieve dual band response for C and X band. Resonator behaviors for different materials are concluded and compared for the same structure. The final design is fabricated over glass-reinforced epoxy material. The fabricated resonator shows a maximum directivity of 5.65 dBi and 6.62 dBi at 5.84 GHz and 8.16 GHz, respectively. The lowest resonance response is less than −20 dB for C band and −34 dB for X band. The resonator is prototyped using LPKF (S63) drilling machine to study the material behavior.

## 1. Introduction

Over the last few years, new composite materials (CM), organic-inorganic (O-I) hybrids, have attracted a significant amount of attention of the research community. New CM are being cited in the present days with better durability, low loss tangent, and high dielectric constant. Material science is becoming popular in radio frequency (RF) technology due to its outstanding behaviour at high frequencies. Different materials are used nowadays for constructing high frequency devices. It is found recently that CM and metamaterial are being used in high frequency technology and researchers are exploring new types of results. High dielectric constant of the substrate material is always desired for high frequency application to have increased amount of directivity and more centralized results. Oxidation behaviour of ZrB_2_ based ultrahigh temperature ceramic composite (UHTC) was tasted in high frequency plasma wind tunnel in [1]. CM were tasted to show the dependence of composition over pressure, enthalpy, and heat flux. A ferrimagnetic compound (Y_3_Fe_5_O_12_) is used as substrate material for a high frequency resonator in [2]. The material is chosen because the Curie temperature of the material is greater than the room temperature which indicates that the magnetic properties of the material remain intact at the operating temperature. The polarization characteristic of the resonating element is changed by changing the direction of the applied magnetic field. To design a nonmetallic resonator resonating at higher frequencies, carbon nanotube (CNT) composite material is used in [3]. Miniaturisation of high frequency device using CM is developing recently. A miniaturization technique for high frequency resonating devices using magnetodielectric and Fe-SiO_2_ sheets is shown in [4]. The placement of the magneto dielectric filling is discussed in their research. A relative permeability of *μ*_*r*_ = 5 and magnetic loss tangent tan*δ*_*m*_ = 0.1 are observed in the magnetodielectric material. Magnetodielectric (MD) material is used in [5] with the measurement of permittivity and permeability of the material. The composition of the material is made using a mixture of Ni-Zn ferrite ceramic and nanosized grains of Ni_0_, _5_Zn_0_, _3_Co_0_, and _2_Fe_2_O_4_ combined. The material composition is complex and the computational result along with the measured result of the designed resonator using the material comes to an agreement. Different DC voltage is applied to the resonator to show the change in capacitance of the material along with the resonance performance. Another MD material is shown in [6] to have a Y-type hexagonal ferrite with the composition Ba_2_Co_2_Fe_12_O_22_ and glass. It is claimed to have better performance than the normal PCB materials for the multi-input multi-output (MIMO) based resonator. Brominated flame retardant-4 (glass-reinforced epoxy) is used as RF and PCB (printed circuit board) material since 1968 by NEMA (National Electrical Manufacturers Association). Epoxy matrix reinforced by woven glass is the basic material for glass-reinforced epoxy. By changing the composition of the fibre glass and epoxy resin, the thickness of the substrate material can be changed and it is direction dependent. The composition has an advantage of not losing their material properties even after few numbers of reuse [7]. The two-material composition has a ratio of 40% and 60% for epoxy resin and fibre glass, respectively [8]. RF products are more likely to generate hit due to high frequency applications and hence burn the substrate material while propagating. Glass-reinforced epoxy is fire resistant (risk of flammability is reduced). Glass-reinforced epoxy material quality control is recently being reviewed by organizations such as UL confirming that, in many cases, glass-reinforced epoxy quality is not kept constant in many industries. By comparing the thermal stability of the glass-reinforced epoxy from different sources, it is shown that some are less stable and others are more thermally stable (higher lead-free processing temperatures) [9]. Bromine, phosphorus, and aluminium hydroxide are chemical means for the glass-reinforced epoxy to be flame retardants. There is a lot of debate about when to use glass-reinforced epoxy instead of high frequency (HF) laminates. The HF laminate materials are said to be good candidate for temperature management inside the board; better impedance matching during the use in RF technologies and electrical performance are comparatively better than glass-reinforced epoxy but it is not significant. But high performance glass-reinforced epoxy has high CTE (coefficient of thermal expansion) *T*_*g*_ (glass transition temperature), high dimensional stability, and robust multilayer fabrication [10] considering the thickness of the glass-reinforced epoxy substrate material. The most important point that makes glass-reinforced epoxy still the best choice is cost effectiveness. Compared to the other substrate materials, glass-reinforced epoxy costs more than 90% less which makes it a great candidate for reproduction of any RF technology. The epoxy resin compressed with the glass fibres gives the substrate an increased amount of dielectric constant of 4.6. The material is anisotropic inherently due to the dense package of weave in one direction [11]. A micromechanical scanner using glass-reinforced epoxy is constructed to have a performance better than conventionally used materials such as silicon micromechanical systems (MEMS) [12].

In this paper, fibre-reinforced plastic characteristic is revised and a description of coupling within the material and at the surface of the material is shown with related equations derivation and graphical view. A planar resonator in microstrip technology is designed using glass-reinforced epoxy material to demonstrate the behaviour at radio frequency and compare it with other materials by using simulation tool. New etching process other than chemical etching in glass-reinforced epoxy is shown using LPKF machine with 0.01 mm accuracy without any crack in the laminate.

## 2. Coupling Mechanism

**Figure 1:**
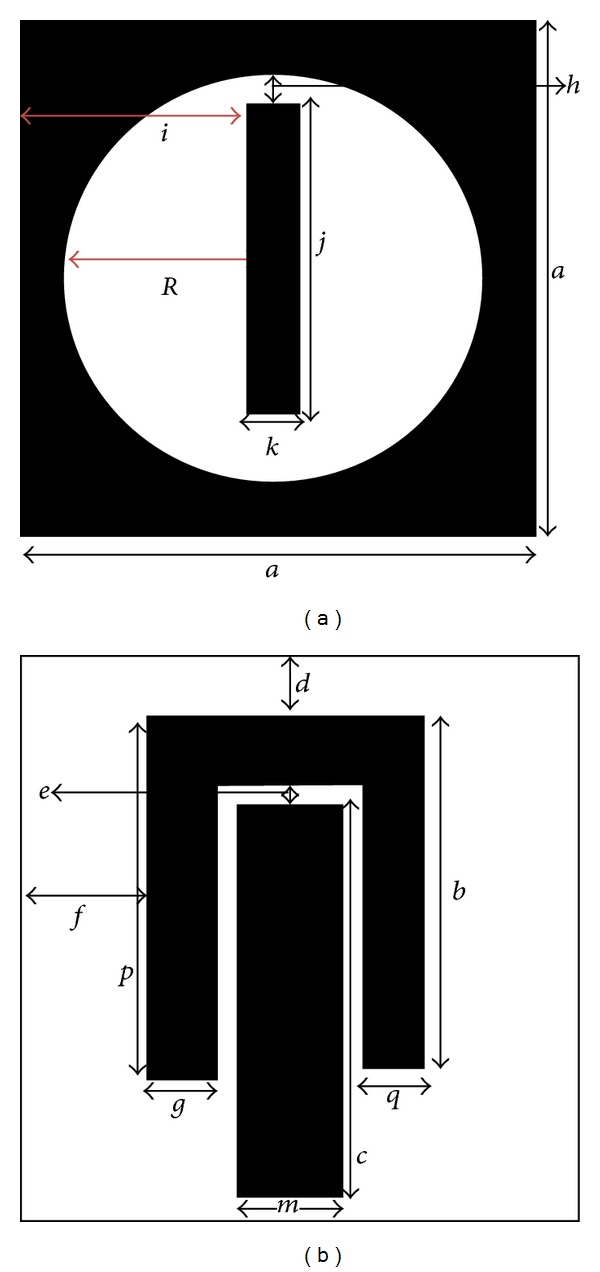
(a) Patch and (b) ground plane of the proposed resonator.

Figure 1 shows the structure of the resonator for characteristics comparison of materials. The parameters are tabulated in Table 2.

Maxwell's equations describe the relation between conductor dielectric and air conductor fields [13] as follows:

(1) $$\begin{array}{c} \overset{}{\underset{s}{\oint }}D\;dS=Q_{\text{fce}},\\ \overset{}{\underset{s}{\oint }}E\;dl=0. \end{array}$$

**Figure 2:**
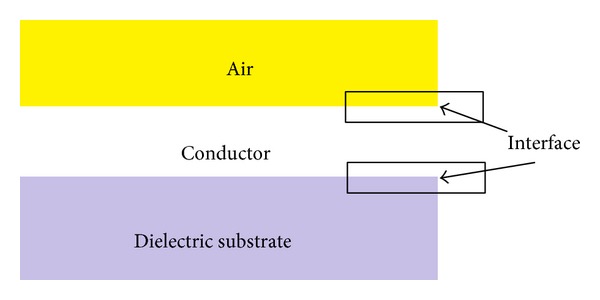
Laired distribution of the resonator substrate material.

Here, *Q*_fce_ is the charge at free space. Copper (Cu) with high thermal and electrical conductivity is considered to have *σ* → *∞* and *ρ*_*c*_ → 0 leading to proper conductor for applications.  Figure 2 shows the relation between dielectric substrate and the conductor. Where the thickness of the conductor tends to be zero, the electric field *E* becomes zero within the conductor. Another electric field that is normal with the conductor is introduced at the interface of the dielectric conductor and air conductor (the electric field calculations is same for both dielectric substrates and air where the dielectric constant for the air is “1”) and is external to the conductor.

**Figure 3:**
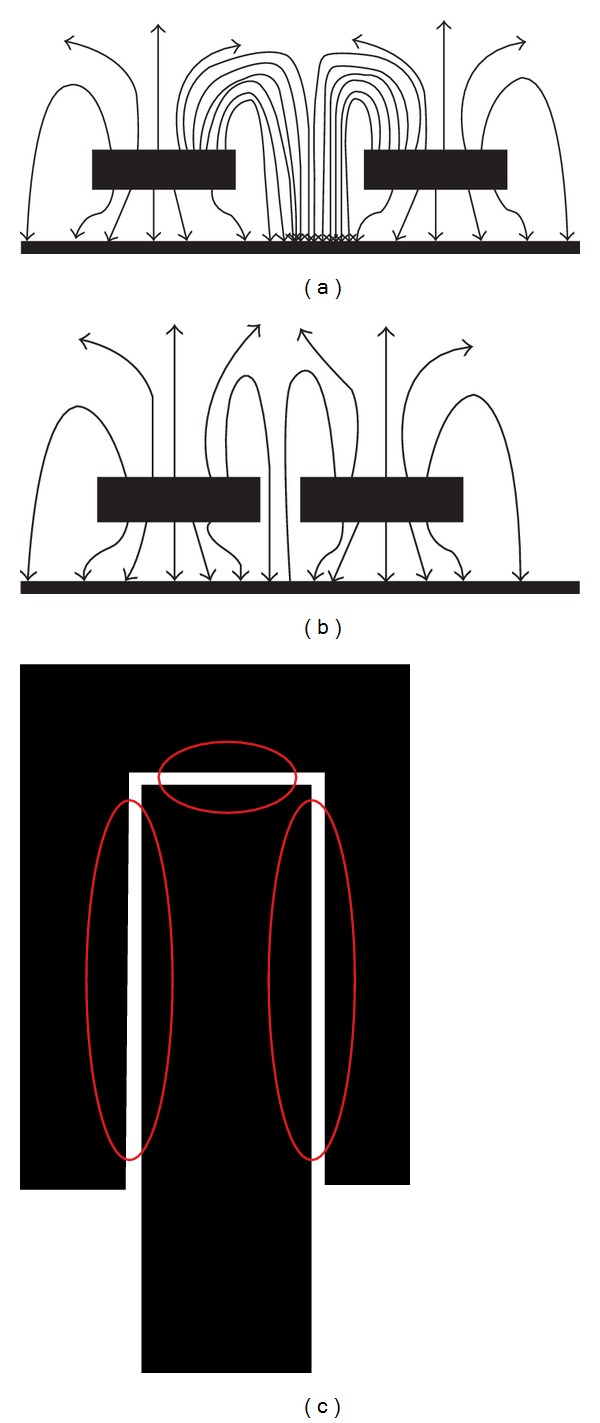
(a) Wide coupling gap; (b) narrowed coupling gap; (c) points of coupling at the patch of the resonator.

The coupling element (passive), which is not connected with the active patch in Figure 3(a), electrically screens (partially) the primary patch. Two structures are divided by a narrow slit resulting in coupling between the elements. The current distribution pattern shown in Figure 10 shows the coupling characteristics between the primary (active) and passive U-shaped element. The even and odd mode impedances characteristic of the resonator controls the coupling between the elements. Analytic formulas are shown below using the even (*Z*_0*e*_) and odd (*Z*_0*o*_) mode impedances. The characteristic impedance (*Z*_0_) is shown to be related with the inductance and capacitance of the coupled structure. The coupled lines with two (electromagnetic consideration) current flow modes because of the coupling current displacement between the conductors and ground plane conductor are specified as differential mode current that is related to the odd mode impedance. The current flow starts from the active patch towards the load matched structure (passive element). Four odd mode capacitances are resulting due to mutual capacitance, the thickness of the coupling conductors (minor capacitance), capacitance between ground planes and coupling lines, and coupling of each other. The inductive values can be said to follow the arguments after considering the magnetic fields. The following equation can be applied for a nondissipative system:

(2) $$\begin{array}{c} Z_{0}=\sqrt{\frac{L\left(\text{inductance}\;\text{per}\;\text{meter}\right)}{C\left(\text{capacitance}\;\text{per}\;\text{meter}\right)}}. \end{array}$$

Even and odd mode characteristic impedances for quarter wavelength matched line can be shown as

(3) $$\begin{array}{c} Z_{0e}=Z_{0}\sqrt{\frac{1+k}{1-k}},\\ Z_{0o}=Z_{0}\sqrt{\frac{1-k}{1+k}}. \end{array}$$

Here,  *k* = 10 − 0.05*C*  (*C* = magnitude of coupling between two lines).

Combining (3), we get

(4) $$\begin{array}{c} Z_{0}=\sqrt{\frac{Z_{0o}}{Z_{0e}}}. \end{array}$$

The values of “*L*” and “*C*” can be found tracking back through the equations provided above. The even and odd mode impedance characteristics calculation for coupled lines is shown in [14] as follows:

(5) $$\begin{array}{c} {\left(Z_{0e}\right)}_{n-1,n}=\frac{1}{y_{0}}\left[1+\frac{J_{n-1,n}}{Y_{0}}+{\left(\frac{J_{n-1,n}}{Y_{0}}\right)}^{2}\right],\\ {\left(Z_{0o}\right)}_{n-1,n}=\frac{1}{y_{0}}\left[1-\frac{J_{n-1,n}}{Y_{0}}+{\left(\frac{J_{n-1,n}}{Y_{0}}\right)}^{2}\right]. \end{array}$$

**Figure 4:**
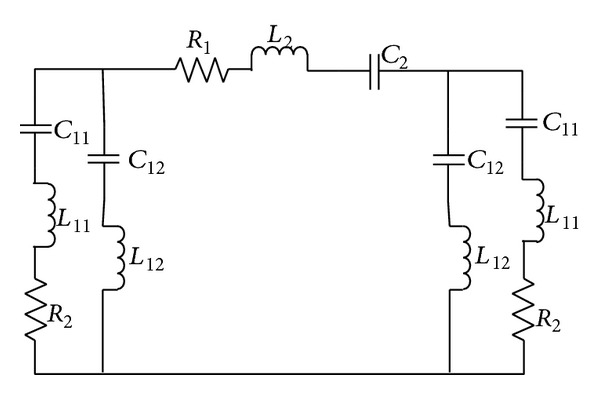
Microstrip coupled line gap equivalent circuit [13].

**Figure 5:**
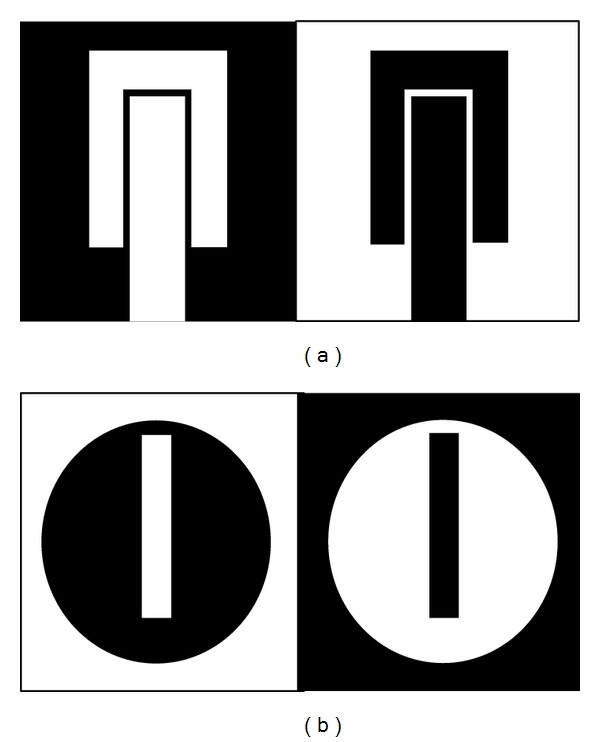
(a) Complementary patch and (b) complementary ground plane.

The even mode impedance increases with the decrement in capacitance and increment in inductance when the gap becomes narrower (0.5 mm for the proposed design).  Figure 3 shows that, with smaller gap between coupled lines, the field dimension tends to decrease between common grounds of the coupled lines. The electric currents propagating through the coupling conductors create magnetic field at the middle of the structure (eddy current effect). Microstrip coupled line gap equivalent circuit is shown in Figure 4. The lumped component values of the circuit can be found using the findings shown in [15]. There is an ignorable amount of effects of the ground planes irregularity over the coupled microstrip lines at the patch. A rectangle microstrip line in the middle of the ground plane circle provides finite ground plane for the patch. The complementary structure for the proposed resonator is shown in Figure 5.

Centering at the middle, a circular slot is cut from the ground plane shown in Figure 1(b); this combination at the ground plane classifies the resonator as aperture type. Babinet's principle explains that the complementary of a circular patch gives the same radiation compared to a circular slot. The equations shown in [16] support the argument as follows:

(2) $$\begin{array}{c} E_{\theta \text{patch}}=H_{\theta \text{complementary}},\\ E_{\varphi \text{patch}}=H_{\varphi \text{complementary}},\\ E_{\theta \text{patch}}=-\frac{E_{\theta \text{complementary}}}{\eta_{0}^{2}},\\ H_{\varphi \text{patch}}=-\frac{E_{\varphi \text{complementary}}}{\eta_{0}^{2}}. \end{array}$$

Here, *E*_patch_ = patch electric field, *η*_0_ = free space intrinsic impedance, *E*_complementary_ = complementary patch electric field, *H*_patch_ = circular patch magnetic field, and *H*_complementary_ = complimentary patch magnetic field.

## 3. Parametric Studies

The structure of the resonator is set to default with the thickness of the substrate, *n* = 1.6 mm. To compare the structure behavior, substrates with different dielectric constants are studied. A total number of four materials are used in the simulation for comparison purpose among materials. The chosen materials are polyester, gallium arsenide, rogers RO 4350B, and glass-reinforced epoxy. Polyester is referred to as polyethylene terephthalate (PET). Synthetic polyester is not biodegradable. From the name gallium arsenide (GaAs), it can be said that it is a mixture of gallium (Ga) and arsenic (As). It is a well-established compound for the use in radio frequency devices, MMICs, and so forth. The chemical compounds used to make GaAs, if varied at a small portion, can affect the purity greatly; hence the process is quite sophisticated. Rogers RO4350B, a conventional high frequency circuit material based on glass-reinforced hydrocarbon/ceramic. The four materials RF characteristic is tabulated in Table 1.

**Figure 6:**
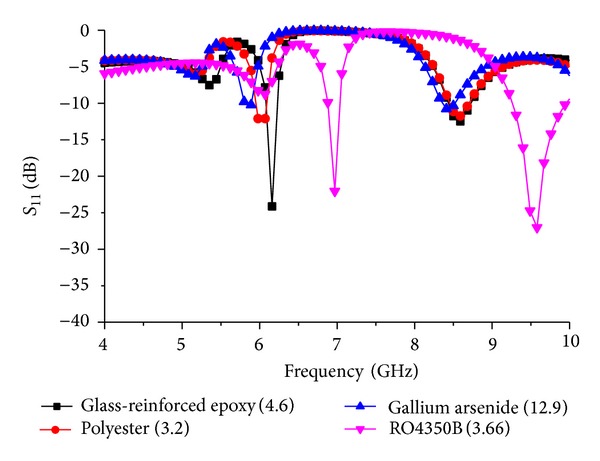
*S*_11_ response for the change in dielectric constant.

Figure 6 shows the response of the resonator for different dielectric constant substrates. Polyester (*ε*_rp_ = 3.2) and glass-reinforced epoxy substrate show similar response at the first resonance frequency which is 0.15 GHz different from each other. As a further matter, gallium arsenide (*ε*_rg_ = 12.9) and glass-reinforced epoxy substrate show similar response at the primary mode resonance frequency which is 0.33 GHz unfamiliar from each other. The second resonance mode of the resonator starts to fade away when the dielectric constant is increased. Glass-reinforced epoxy gives a valid response for the second resonance mode. Rogers RO4350B substrate (*ε*_RO_ = 3.66) a popular [17, 18] substrate is considered in Figure 6. The first resonance mode increases at an amount of 0.78 GHz from the default glass-reinforced epoxy substrate response. It can be observed that the substrate material of the resonator plays part in the frequency resonance response which gives different results for different substrate materials. The impedance matching at the input/output is the key point for resonance. In this case, 50 Ω impedance matching is chosen for simulation over the materials described. Later, the prototype is fabricated using a 50 Ω SMA connector to validate the simulation result and measured result of the resonator.

**Figure 7:**
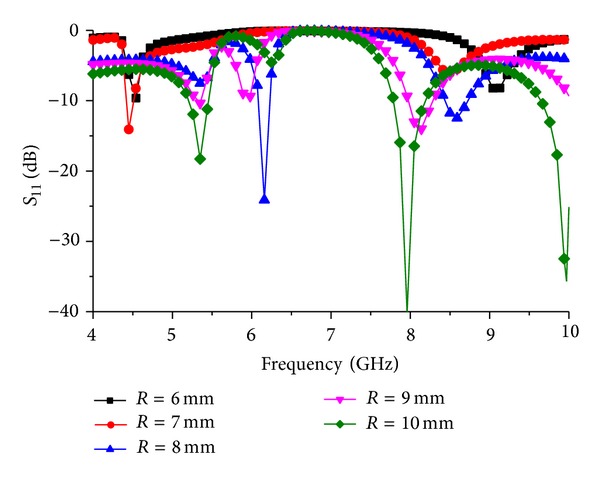
Resonator resonance response with changed radius of circular slot.

Changing the ground plane circular slot radius at the ground plane, the response of the frequency resonance can be shifted.  Figure 7 shows the frequency response for finite change in “*R*.” The circle radius is limited to *R* = 6 mm, because at this value the rectangle at the middle of the ground plane unites with the outer shell of the circular slot and the first mode resonance is shifted to 4.51 GHz. However, the second mode resonance fades away. For *R* = 7 mm, the response of the resonator stays almost the same compared to the resonance response for *R* = 6 mm. For *R* = 9 mm, the first resonance response fades away leaving a narrow response at 5.89 GHz. The second mode resonance shifts to 8.05 GHz with a bandwidth of 330 MHz. At *R* = 10 mm, total resonance response of the resonator shows harmonic behavior. The first mode resonance moves at 5.35 GHz with a bandwidth of 120 MHz and the second mode resonance moves at 7.96 GHz. A third mode resonance is introduced at 10 GHz.

**Figure 8:**
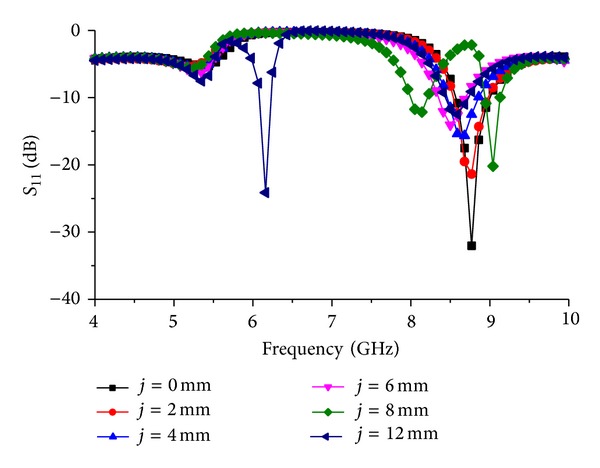
Resonator resonance response for change in length “*j*.”

Figure 8 shows the resonance response of the resonator for the change in the length “*j*” where “*j*” is the length of the rectangle situated at the middle of the ground plane. From the figure, it can be seen that the first mode resonance is more dependent on the length of the rectangle. After optimizing the placement of the rectangle manually, the length “*j*” is changed to observe the change in resonance response. The first mode resonance is fully missed when length “*j*” is decreased.

**Figure 9:**
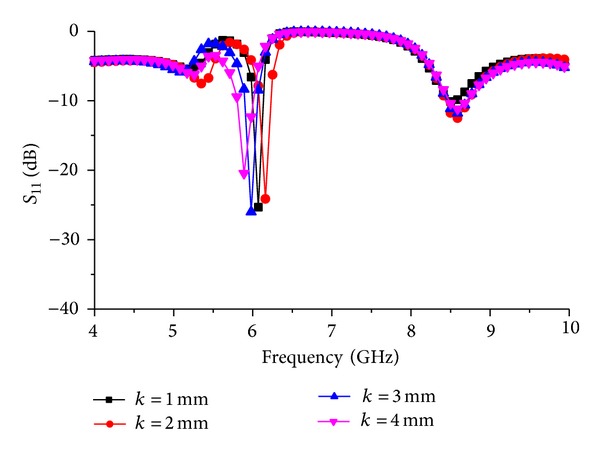
Resonator resonance response for change in width “*k*.”

As microstrip rectangle in the middle of the ground plane plays an important role, the width “*k*” is changed to observe the resonator response shown in Figure 9. The response is shown for the glass-reinforced epoxy substrate to observe the material behavior.

**Figure 10:**
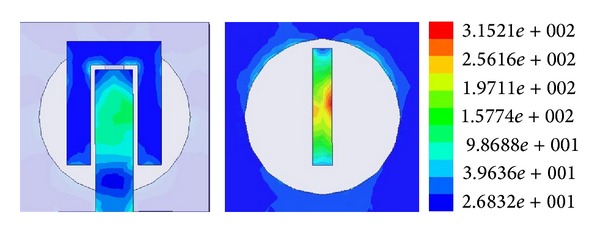
Current distribution pattern of the resonator.

The current distribution pattern in Figure 10 shows that, at 6.18 GHz frequency, the rectangle situated at the middle of the ground plane plays an important role. The current density is at its peak at the outer edge of the rectangle. Minor perturbations can be observed at the active patch. However, the U-shaped coupler shows light response to the current distribution pattern. Tuning the length and width of the rectangle in the middle of the ground plane, the resonance response can be varied.

## 4. Optimization Using RCPSO Algorithm

RCPSO is a particle swarm optimization (PSO) based algorithm developed for optimizing the directivity of microstrip resonator. With enough computation time given, this optimization algorithm can develop resonator directivity characteristics [19]. Premature convergence and dimensionality are the drawbacks of PSO. Using the multistart algorithm, searching areas are broke into 2D and 3D pieces including the thickness of the substrate and substrate dielectric constant. Hence, the premature convergence problem is ignored. The dimensional problems in RCPSO are solved using k-dimensional breakdown system. The optimization process is carried on until a fixed number of iterations or until the goal of optimization is met. Following the equation below, PSO updates the position of the particle:

(7) $$\begin{array}{c} v_{i+1}=wv_{i}+\varphi_{1}r_{1}\left(d_{\text{localbest}}-d_{i}\right)+\varphi_{2}r_{2}\left(d_{\text{globalbest}}-d_{i}\right),\\ d_{i+1}=d_{i}+v_{i+1}, \end{array}$$

where *w* = coefficient of the inertia, *r*_1_, *r*_2_ = random values uniformly distributed between zero and one, *v*_*i*_ = velocity of the particle at iteration “*i*”, *d*_*i*_ = dimensions of a particle at iteration “*i*,” and *φ*_1_,   *φ*_2_ = cognitive and social coefficients, respectively.

The modified PSO is designed specifically for aperture type resonator optimization. Commercially available IE3D and MATLAB software are used to initiate the optimization. Without considering the substrate thickness and dielectric constant, 9 dimensions of the resonator patch were optimized. The optimization process is initiated holding two random dimensions as constants and other dimensions are set free to move within the substrate dimension. When the 9-generation cycle is complete, the algorithm reinitiates the population by making the two constants as free dimensions and two other dimensions are set to be constant. The choice of constant is random. The resonator dimension before optimization and after optimization is given in Table 2.

The average directivity of the resonator is optimized; hence, it was the fitness function for the maximization problem.  Figure 15 shows the directivity improvement over generations.

## 5. Resonator Measurement

**Figure 11:**
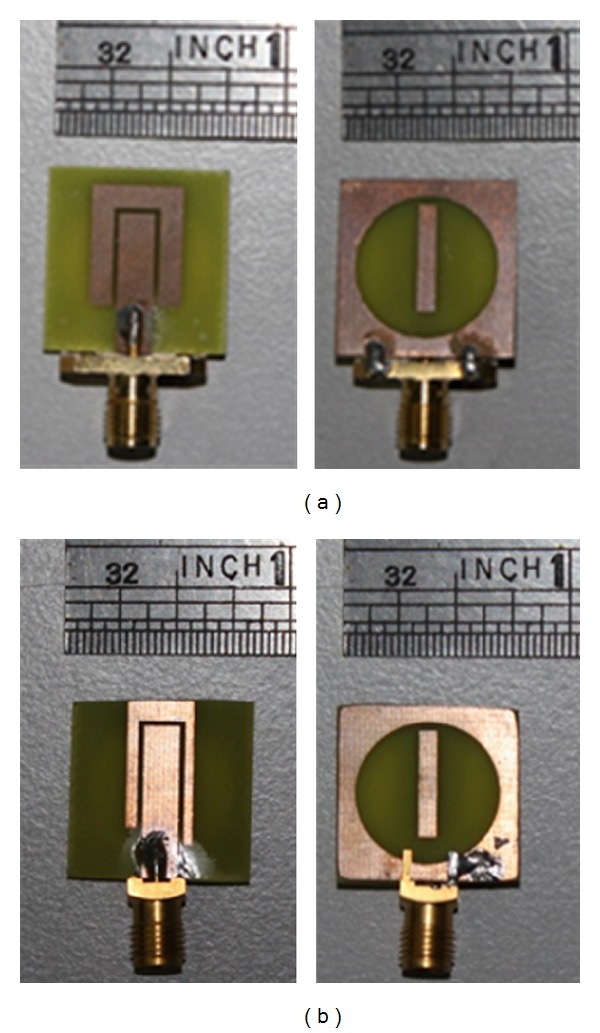
Resonator (a) before optimization and (b) after optimization.

Agilent power network analyser of model number E8358A is used to measure both resonators as shown in Figure 11. A 4.5-meter by 3.5-meter anechoic chamber is used to measure the directivity and polarization of the resonator. “Three resonator” measurement technique is followed to get the resonator characteristics. For the far field measurement at 5.5 GHz, the minimum far field distance can be calculated as *d*_*f*_ ≫ 2*λ*. A three-meter distance between the resonators in the chamber serves as far field in this case. Two linearly polarized horn antennas of model number SAS 571 and the resonator under test (RUT) serve as three resonators in this technique. Two horn antennae are measured for directivity response within them. After that, the RUT directivity is measured in reference to both horn antennae. Friis transmission equation for the directivity measurement can be demonstrated as

(8) $$\begin{array}{c} G_{i}\;\text{dB}+G_{j}\;\text{dB}=20\;\text{lo}{\text{g}}_{10}\left(\frac{4\pi R}{\lambda }\right)+10\;\text{lo}{\text{g}}_{10}{\left(\frac{P_{r}}{P_{t}}\right)}^{k}. \end{array}$$

Here, *i* = 1, 2, 3, *j* = 1, 2, 3,  *i* ≠ *j*, and *k* = 1, 2, 3. *R* = distance between the measuring resonators, *P*_*r*_ = received power, *G* = directivity of the resonator, and *P*_*t*_ = transmitted power.

## 6. Results and Discussion

The technology for PCB prototyping is advancing exponentially with the time. Thanks to LPKF, a prototyping machine company based in Germany, as they have developed a PCB prototyping machine that can prototype a dual sided PCB laminate of marginal length, width, and thickness. This machine can automatically etch out the unwanted copper layer from the RF laminate by using fast spin driller. The accuracy of the machine is as narrow as 0.01 mm. For both sides prototyping, a camera is attached with the driller so that calibration can be done before etching out both layers. The machine uses software named LPKF circuit pro to give command using computer. For fast spin technology, if a hole is drilled on a glass-reinforced epoxy laminate, the glass-reinforced epoxy will not break down at the side of the hole or whatsoever. By using this machine, glass-reinforced epoxy laminate material can easily be integrated with any type of RF module and any design specification using microstrip lines over glass-reinforced epoxy laminate is possible.  Figure 16 shows the machine used for the fabrication of the resonator on glass-reinforced epoxy material.

Figure 11 shows the fabricated resonator using glass-reinforced epoxy substrate with a thickness of 1.6 mm and a dielectric constant of 4.55. Figures 11(a) and 11(b) show the resonator fabricated before optimization and after optimization, respectively. Comparing the figures, there is a change at the U-shaped passive element. The ground plane contains no change in terms of dimension because the ground plane is left out of optimization.

**Figure 12:**
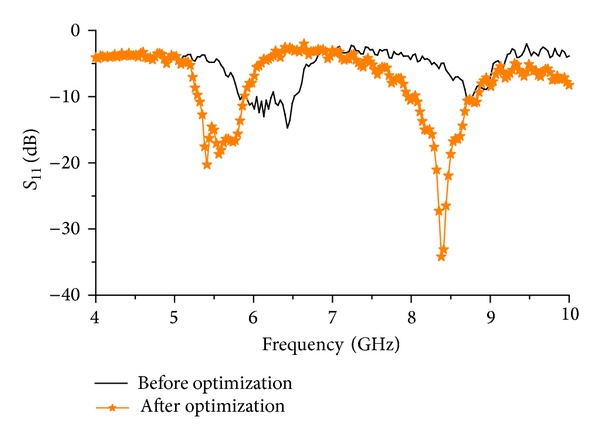
Measured *S*_11_ response of the resonators.

The measured resonance responses for both resonators are shown in Figure 12. The first resonator (before optimization) −10 dB bandwidth is 5.89 GHz to 6.52 GHz with a notch at 6.25 GHz. The notch degrades the total bandwidth performance of the resonator. The second mode resonance bandwidth starts from 8.71 GHz till 8.77 GHz. The second mode resonance is very narrow compared to the simulation result. By optimization technique, the desired band is achievable in glass-reinforced epoxy substrate. Though glass-reinforced epoxy is limited to operating at lower frequency, this resonator shows stable resonance performance even at higher frequencies.

**Figure 13:**
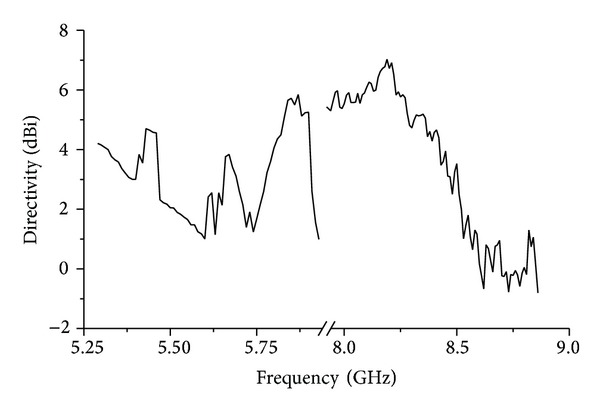
Peak directivity of the optimized dual band resonator.

**Figure 14:**
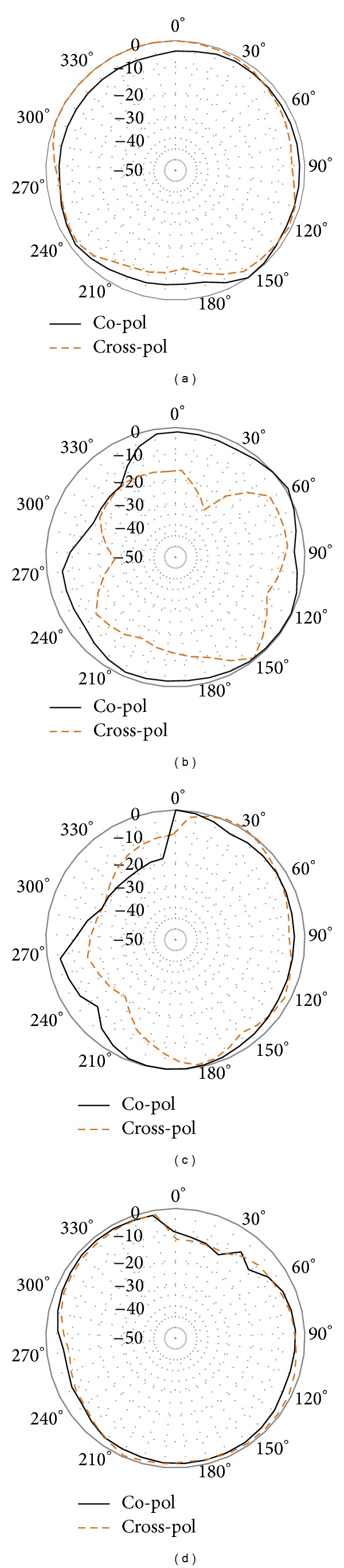
Normalized radiation pattern of the resonator at (a) 5.5 GHz (*E*-plane), (b) 8.3 GHz (*E*-plane), (c) 5.5 GHz (*H*-plane), and (d) 8.3 GHz (*H*-plane).

**Figure 15:**
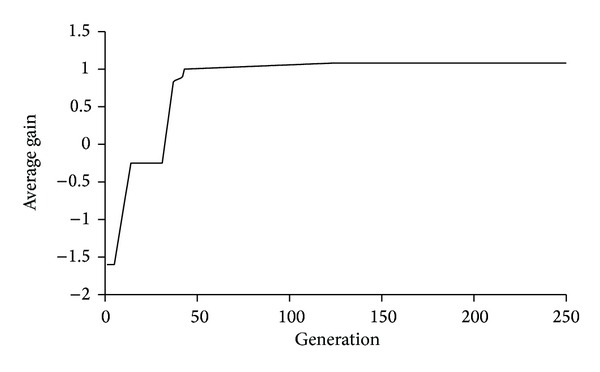
Average directivity (gain) increment after 246 generations (taken from Matlab).

**Figure 16:**
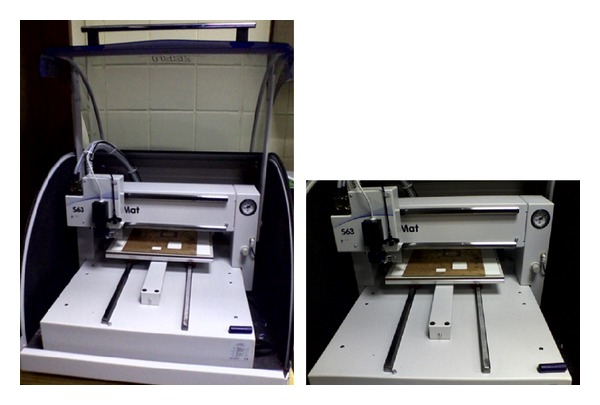
LPKF (S63) PCB prototyping machine.

Figure 12 also includes the resonance response of the optimized resonator. It can be observed from the figure that the bandwidth of the optimized antenna is increased along with the increment in the directivity. A bandwidth of 0.6 GHz is achieved from 5.29 GHz to 5.89 GHz at the first mode resonance and a bandwidth of 0.93 GHz is achieved at the second mode resonance starting from 7.93 GHz to 8.86 GHz. Less than −20 dB return loss is found at the first mode resonance and less than −34 dB second resonance response is found. The measured directivity of the optimized resonator is shown in Figure 13. An average directivity of 3.2 dBi is achieved at the frequency starting from 5.29 GHz to 5.93 GHz. At 5.84 GHz, the peak directivity can be observed to be 5.65 dBi. A peak directivity of 6.62 dBi is achieved at 8.16 GHz frequency with a wide bandwidth response. The average directivity of the second mode resonance is 4.3 dBi starting from 7.93 GHz to 8.68 GHz. The normalized radiation patterns at the first and second mode resonance are shown in Figure 14. At 5.5 GHz, the radiation pattern behaviour is observed to be omnidirectional. For the second mode resonance at 8.3 GHz, the radiation pattern seems to behave like a directional resonator compared to the first mode resonance.

## 7. Conclusions 

Coupling mechanism for microstrip technology is described in this paper. Glass-reinforced epoxy material characteristic is revised. The advantage of using glass-reinforced epoxy material for RF application is described. The use is shown by designing a resonator on glass-reinforced epoxy laminate. Simulation is carried out by using different substrate for the same microstrip resonator design. The simulation results are compared. A U-shaped parasitic element is introduced covering the active patch body of the resonator. The dimension of the patch is optimized using RCPSO algorithm. To compare the results of before and after optimization, the resonators were fabricated and measured. U-shaped parasitic element can be optimized to tune the resonance frequency of the resonator. The resonator shows a maximum directivity of 5.65 dBi at 5.84 GHz and 6.62 dBi at 8.16 GHz after optimization. A large array using fire resistant, robust glass-reinforced epoxy laminate material for C and X band application is proposed for the future work. As the material glass-reinforced epoxy itself is cost effective, a large array for high gain in C band and X band application is a feasible choice.

## Figures and Tables

**Table 1:** Characteristics of polyester, gallium arsenide, glass-reinforced epoxy, and rogers RO 4350B (from HFSS “high frequency structural simulator”).

	Polyester	Gallium arsenide	Glass-reinforced epoxy	RO 4350B
Relative permittivity (*ε*)	3.2	12.9	4.6	3.66
Relative permeability (*μ*)	1	1	1	1
Dielectric loss tangent (tan⁡*δ*_*m*_)	0.003	0	0.02	0.004
Mass density	0	5320	1900	0
Fire resistant	Yes	No	Yes	No
Durability	Used in cloths	Durable	Durable	Durable
Cost	Low cost	*Unknown *	Very low cost	High cost
Availability	Available	On order (toxic product)	Available	Available
Found with preloaded copper laminate	No	No	Yes	Yes

**Table 2:** Design specification of the proposed resonator.

Resonator dimension and substrate details	Before optimization (in mm)	After optimization (in mm)
Substrate	Glass-reinforced epoxy	Glass-reinforced epoxy
Relative permittivity of the substrate (*ε*_*r*_)	4.55	4.55
Thickness of the dielectric substrate (*n*)	1.6	1.6
Ground plane (*a* × *a*)	20 × 20	20 × 20
Hands length of the coupling element (*b*)	13.5	16.2
Hands length of the coupling element (*p*)	13.5	14.3
Length of the primary patch (*c*)	15	16.9
Distance from the boundary to coupling element (*d*)	2	0.15
Gap between primary and coupling elements (*e*)	0.5	0.61
Distance from the boundary to the hand of the coupling element (*f*)	5	6.2
Width of the coupling element (*g*)	2.5	1.2
Width of the coupling element (*q*)	2.5	1.23
Width of the primary patch (*m*)	4	4
Distance from the circular slot to the rectangle (*h*)	1	1
Width of the rectangle (*k*)	2	2
Length of the rectangle (*j*)	12	12
Distance from the boundary to the rectangle (*i*)	9	9
Radius of the circular slot (*R*)	8	8
